# Two-Stage Clustering (TSC): A Pipeline for Selecting Operational Taxonomic Units for the High-Throughput Sequencing of PCR Amplicons

**DOI:** 10.1371/journal.pone.0030230

**Published:** 2012-01-11

**Authors:** Xiao-Tao Jiang, Hai Zhang, Hua-Fang Sheng, Yu Wang, Yan He, Fei Zou, Hong-Wei Zhou

**Affiliations:** 1 Department of Environmental Health, School of Public Health and Tropical Medicine, Southern Medical University, Guangzhou, Guangdong, China; 2 NetworkCenter, Southern Medical University, Guangzhou, Guangdong, China; Argonne National Laboratory, United States of America

## Abstract

Clustering 16S/18S rRNA amplicon sequences into operational taxonomic units (OTUs) is a critical step for the bioinformatic analysis of microbial diversity. Here, we report a pipeline for selecting OTUs with a relatively low computational demand and a high degree of accuracy. This pipeline is referred to as two-stage clustering (TSC) because it divides tags into two groups according to their abundance and clusters them sequentially. The more abundant group is clustered using a hierarchical algorithm similar to that in ESPRIT, which has a high degree of accuracy but is computationally costly for large datasets. The rarer group, which includes the majority of tags, is then heuristically clustered to improve efficiency. To further improve the computational efficiency and accuracy, two preclustering steps are implemented. To maintain clustering accuracy, all tags are grouped into an OTU depending on their pairwise Needleman-Wunsch distance. This method not only improved the computational efficiency but also mitigated the spurious OTU estimation from ‘noise’ sequences. In addition, OTUs clustered using TSC showed comparable or improved performance in beta-diversity comparisons compared to existing OTU selection methods. This study suggests that the distribution of sequencing datasets is a useful property for improving the computational efficiency and increasing the clustering accuracy of the high-throughput sequencing of PCR amplicons. The software and user guide are freely available at http://hwzhoulab.smu.edu.cn/paperdata/.

## Introduction

Determining 16S rRNA gene tags using pyrosequencing has become an important tool for studying microbial diversity. This approach has led to many interesting findings relating to both human and environmental microbial habitats. For instance, chronic metabolic diseases such as obesity and diabetes are potentially related to gut microbiome diversity [1], [2], [3], and unexpectedly high bacterial diversity is found in aquatic environments [4], [5]. Recently, we developed a barcoded Illumina paired-end sequencing (BIPES) method for determining 16S rRNA tags using the Illumina HiSeq 2000 [6]. Illumina platforms are able to obtain millions of tags relatively cost-effectively, but they generates new problems for analysis [6], [7], [8], [9], [10]. Within the bioinformatics pipeline, clustering tags into operational taxonomic units (OTUs) according to sequence similarity is a rate-limiting step for analyzing microbial diversity, for which the computational demand increases geometrically with sequencing read number [11]. In addition to the Illumina platforms, the upgraded 454 Life Sciences Titanium instrument also results in millions of reads per run, which also raises new computational issues.

The present study therefore focused on developing a pipeline for choosing OTUs for the high-throughput sequencing of PCR amplicons. We focused on three major issues, namely accuracy, time, and peak memory, to develop the pipeline. Sun et al. [12] identified the problem of the biased OTU estimation of clustering methods. They found that multiple sequence alignment was less accurate than pairwise alignment for calculating distances for a large number of sequences, and they reduced the OTU number using the pairwise Needleman-Wunsch (NW) distances. In addition to the sequence-alignment algorithms, Huse et al. [13] found that clustering sequences by average linkage (AL) was more robust than complete linkage (CL) or single linkage (SL) in terms of reducing biases. Furthermore, they developed a single-linkage preclustering (SLP) method that preclustered sequences at the 0.02 distance using SL before the formal clustering of tags at the 0.03 distance using AL, thereby mitigating the effect of ‘noise’ sequences that might be produced by PCR and sequencing errors.

While the above methods increase clustering accuracy, they do not scale up well for large datasets. For instance, the number of pairwise NW alignments increases geometrically, which may require tens of thousands of CPU hours if analyzing millions of sequences. Moreover, the distance matrix file, which needs to be loaded into the memory for AL clustering, would be larger than 1000 GB. In ESPRIT, the calculation demand is reduced by prescreening tags using the Kmer distance, and a sparse matrix is produced to reduce the matrix file size. In addition, the authors of ESPRIT developed an hcluster program, which can perform CL clustering without loading the matrix file into memory [12]. However, ESPRIT still does not scale up well for calculating a large number of pairwise NW distances, and only the CL using hcluster had a relatively low memory demand. The authors of ESPRIT claim that their improvements of ESPRIT-Tree and ESPRIT-Forest will resolve the upscaling problems [14], [15]. In addition to these methods, another new algorithm, called UCLUST, is highly efficient in clustering large datasets [16]; this algorithm will be included in the comparison in the present study.

Rather than developing new alignment and clustering algorithms, the present study aimed to improve the computational efficiency from a different angle: the characteristics of the sequencing dataset. High-throughput sequencing results for PCR amplicons have a unique characteristic: they usually have a small fraction of abundant sequences and a majority of rare sequences, whether they are amplified from mock libraries with a known number of templates or from real community samples with diverse templates [4], [13]. In particular, singletons plus doubletons usually make up approximately 70–80% of the total number of unique tags, meaning that only ∼20% of all tags are present in three or more copies [13], [17]. Accordingly, we separated tags into two groups and clustered them sequentially in a two-stage clustering (TSC) pipeline to reduce the computational demand for calculating the large matrix of pairwise NW distances. The results suggest that the TSC pipeline not only increases the efficiency of OTU selection but also improves accuracy by reducing the spurious OTUs incurred by low-abundance ‘noise’ sequences and leads to comparable or improved performance in beta-diversity comparisons of microbial communities.

## Methods

### The TSC algorithm

**Figure 1:**
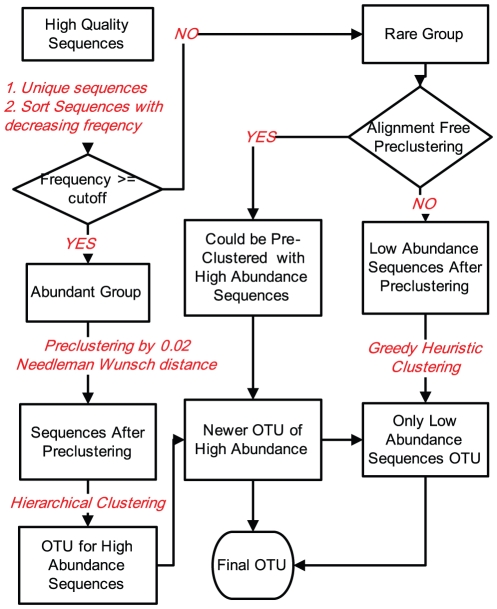
A workflow of the TSC pipeline.

We used C++ to program the TSC pipeline. The program is standalone software and can run on Linux systems directly after download. The workflow of the TSC pipeline is shown in Fig. 1.

#### Dividing tags into two groups

The first step in the TSC pipeline is to divide tags into two groups according to their abundance. The cutoff value can be any number greater than or equal to 1. The cutoff value 1 is used only for testing the algorithm, not for analyzing real samples. The default cutoff value is 3 because many singleton or doubleton sequences might be sequencing or PCR errors [13], [17], [18]. We obtained unique tag sequences from the original data, and the tags were sorted from high to low frequencies and divided into two files according to the cutoff value.

#### First-stage clustering

We used a hierarchical clustering algorithm with pairwise NW distances to cluster the tags in the abundant tag file. An algorithm similar to ESPRIT, but OpenMP enabled, was used to calculate pairwise NW distances of sequences in the abundant tag file. We set options −g −10 −e −1 −x, and the maximum value for Kmer distance was 0.5 for TSC. All of these parameters are the same as the defaults in ESPRIT and can be changed by the user. We then performed one round of the NW alignment-dependent preclustering step (preclustering 1) similar to, but more stringent than, that in the SLP pipeline [13]. The rationale is that some tag sequences in the abundant group might be ‘noise’ sequences produced as a result of PCR-induced mutation or sequencing errors. These sequences theoretically are highly similar to, but less abundant than, their parent tags [13], [18], and they should be merged into their corresponding parent tags rather than participate the OTU clustering [13]. Because the first-stage clustering in the TSC pipeline only treats the abundant tags, we only performed 1 round of preclustering, which is different from SLP and includes two rounds of SL for OTUs with fewer than 10 unique tags [13]. All tags in the abundant group were sorted from high to low abundance. We began the search with the most abundant sequence. If any sequence with lower frequency had a pairwise NW distance equal to or less than 0.02 from the highest frequency sequence, the lower-frequency tag was removed from the list, and the frequency of the highest tag was increased by 1. Next, the second-most abundant sequence served as the seed sequence, and tags merged into the highest seed were not compared again. The remaining seed sequences after the final denoising comparison were clustered with pairwise NW distances. The clustering method at this step can be selected for CL, AL, or SL algorithms, and the resulting OTUs were labeled as highly abundant OTUs (HAOTUs).

#### Second-stage clustering

We used a greedy heuristic algorithm for assigning rare tags into OTUs. We first used an alignment-free preclustering algorithm (preclustering 2) to improve the efficiency. Each pair of sequences was directly compared from the 5′ to the 3′ end one base at a time, and all mismatches were counted. If the distance calculated directly using the mismatch number was lower than the expected distance, the two sequences could be merged directly.

Subsequently, residual rare sequences were sequentially clustered. We first calculated the Kmer distance of an enquiring rare tag against sequences already in OTUs and sorted them accordingly. This Kmer calculation was the memory-limiting step for the TSC pipeline. The pairwise NW distance was calculated for 10 (a changeable parameter) sequences with the lowest Kmer distances with the new enquiring rare tag. If any pairwise NW distance fit the input threshold, for example 0.03, the program calculated NW distances for the next 10 pairs of sequences and repeated the process until all 10 NW distances were larger than the threshold. With these NW distances, there were a total of 5 possibilities for clustering according to the type of abundant or rare tags with NW distances from the new rare tag equal to or less than 0.03. First, if the new rare tag showed NW distances larger than 0.03 with any tags, it formed a new low-abundance OTU (LAOTU). Second, if the new rare tag had less than 0.03 NW distance from tags from only one OTU, it was assigned into the specific OTU whether the OTU had high or low abundance. Third, if the new rare tag fit the distance threshold with abundant tags from more than one HAOTU, it was linked with the tag with the highest abundance and assigned into the same HAOTU. The rare tag did not link these HAOTUs, although all of them harbored tags showing 0.03 or shorter pairwise NW distances from the new rare tag. Fourth, if the new rare tag was connected with rare tags from more than one LAOTU, it grouped all LAOTUs into one LAOTU. Finally, if the rare tag fit distance thresholds with tags from both HAOTUs and LAOTUs, we grouped all LAOTUs together and merged all of them into the HAOTU with the most abundant tag. In brief, we allowed rare tags to group LAOTUs but not HAOTUs.

### The computing environment

We used a desktop computer with an Intel Core™ i7-980X processor (6 cores), 12 GB memory, and a 500 GB hard disk. The operating system was Linux with OpenMP enabled.

### The operational parameters for the methods compared in the present study

#### TSC

The default parameters were −g −10 −e −1 −a 1 −b −1 −k 6 −f 0.5 −d 0.03 −n 10 −x 1 −s 10 −m [al/cl/sl]. Detailed explanations of these parameters can be found in the user's guide.

#### ESPRIT

We used kmerdist with default parameters; for needledist, we used −g 10 −e 1 and added −x.

#### SLP-PW-AL

We used ESPRIT needledist to calculate the pairwise NW distance. The parameters were the same as those in ESPRIT. We used the default parameters for SLP, and we used mothur to carry out AL clustering with the pairwise NW distance calculated by ESPRIT.

#### Mothur

for CL and SL, we used cutoff = 0.10 in dist.seqs, whereas for AL, we used cutoff = 0.42 in dist.seqs to generate enough distances to obtain an AL of 0.03 distance in mothur. Default settings were used for the remaining parameters.

#### UCLUST

First, we sorted the tags by abundance and ran them with the −usersort −id 0.97 −iddef 3 −nofastalign. We used the OTU with 97% identity in UCLUST for the comparison with the 0.03 distance OTUs in other methods.

## Results and Discussion

### TSC estimates of the expected OTU number using mock library data

We first focused on the accuracy of the clustering result. We used a dataset named clone43_97up, which was initially reported by Huse et al. for evaluating and developing the SLP pipeline [13]. This dataset was generated using 454 sequencing of V6 tags amplified from a clone library consisting of only 43 taxa, from which sequences with NW distances greater than 0.03 from their initial templates were further removed. The dataset contained 193,958 total and 4,034 unique tags, which ideally should be clustered into 43 OTUs at the 0.03 distance [13].

**Figure 2:**
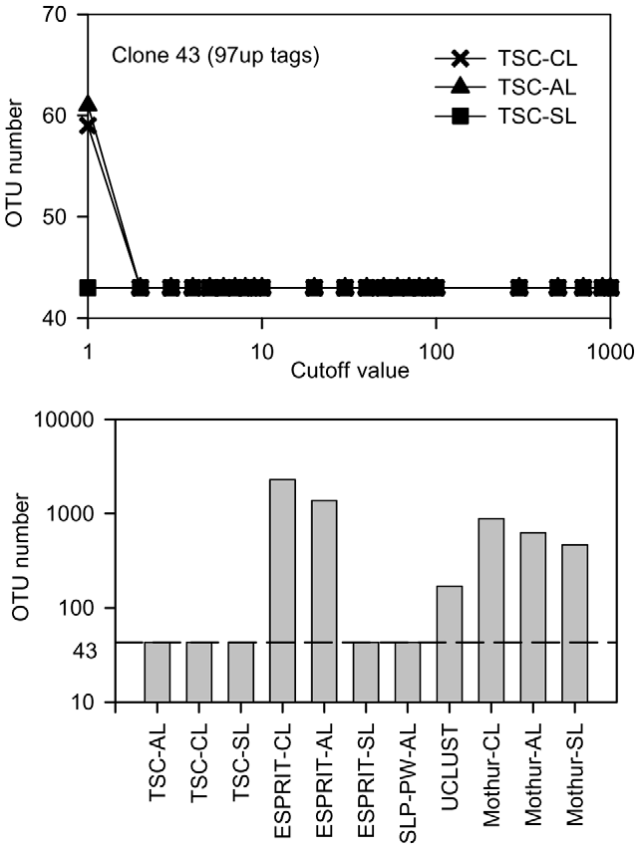
Clustering of the clone43_97up data. (A) The clustering results at different cutoff values. (B) The clustering results using different methods. The cutoff value for TSC was 3.

When the cutoff value was set as 1, all tags were put into the abundant group, and the TSC pipeline only ran the first-stage clustering, which was similar to the approach of SLP-PW-AL except that a more stringent preclustering was employed. Only the TSC-SL method obtained 43 OTUs, whereas the other two methods showed excessive OTU estimation (Fig. 2). In the SLP report, Huse et al. suggested that SL caused underestimation, CL lead to overestimation, and AL was preferable because it obtained the expected 43 OTUs using SLP-PW-AL [13]. In our study, the TSC-AL (cutoff 1) obtained more than 43 OTUs because TSC only ran 1 round of sequential SL preclustering using the 0.02 distance, whereas the SLP performed 2 rounds for OTUs with fewer than 10 unique tags (Methods).

With the cutoff value of 2, the TSC pipeline showed the expected 43 OTUs (Fig. 2). At a cutoff of 2, tags with 2 or more copies were first clustered and grouped into the expected 43 OTUs using any of the CL/AL/SL algorithms; at the second stage, singleton tags were clustered into the existing 43 OTUs using the greedy heuristic SL algorithm, and no additional OTUs formed from these singleton tags. In other words, some singleton tags caused spurious OTU numbering using CL/AL algorithms at the cutoff value of 1. In addition to OTU number, we manually verified that the 43 OTUs were correctly represented by the original 43 template sequences.

Among our tested methods, only TSC (with a cutoff of 2 or higher), ESPRIT-SL, and SLP-PW-AL obtained the expected 43 OTUs; all other methods resulted in much higher values (Fig. 2 and Table 1). A direct comparison of the OTU numbers obtained from different methods might be misleading in terms of accuracy because different alignment methods, e.g., multiple sequence alignment vs. pairwise alignment, and clustering algorithms including CL/AL/SL were employed. For the clone43_97up dataset, the tags were screened, and only those sequences with a 0.03 or smaller pairwise NW distance to the original 43 templates were included. Therefore, these tags should be expected to be grouped into 43 OTUs, represented by the 43 initial templates, at the 0.03 distance, as demonstrated with SLP-PW-AL [13]. Our results show that the TSC pipeline can reproducibly group these tags into 43 OTUs with a large window for selecting the cutoff value. In addition to the clone43 dataset, we tested the method using the clone 90 dataset as described by Quince et al. [19], and the TSC pipeline obtained the expected 30 OTUs at a cutoff value of 3, demonstrating that the TSC pipeline can be used for various datasets.

**Table 1:** A comparison of different methods.

	TSC-CL	TSC-AL	TSC-SL	ESPRIT-CL	ESPRIT- AL	ESPRIT- SL	SLP-PW-AL	UCLUST	Mothur-CL	Mothur-AL	Mothur-SL
***clone43_97up dataset***											
OTU number	43	43	43	2289	1383	43	43	170	888	627	466
***Costello day 3 dataset***											
OTU number	5002	4993	4939	9552	7733	4894	6420	9321	16817	14892	11585
Execution time(s)	370	371	369	43243	43653	43213	45583	38	3073	19750	3073
Peak memory (MB)	185	185	185	152	1419	152	2200	144	834	8600	834

The running parameters for TSC were at cutoff value of 3 with 10 CPU threads.

### TSC significantly reduces computing time and peak memory usage

To demonstrate the efficiency of the TSC pipeline for analyzing real community data, we used the Costello dataset (ERA000159), which was the first report on the microbial diversity in 12 individual humans at four sampling times [20]. Within this dataset, we used the ‘day 3’ dataset, which contained 116,736 total tags with 38,572 unique sequences sampled at day 3, to compare different algorithms because the full Costello dataset was computationally too expensive to analyze on our desktop computer using ESPRIT, SLP-PW-AL, and Mothur-AL.

**Figure 3:**
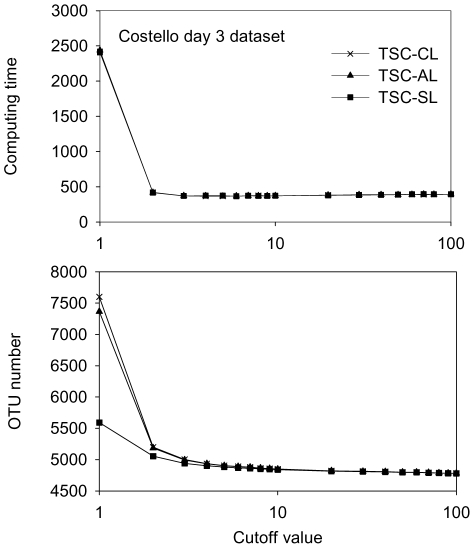
TSC clustering of the Costello day 3 dataset. (A) The computing time at different cutoff values. (B) The OTU number at different cutoff values using the CL, AL and SL algorithms.

Separating tags into 2 groups significantly reduced the computational demand. The runtime for the Costello day 3 dataset with a cutoff value of 1 (1 group) was approximately 2,500 s with 10 CPU threads, which significantly decreased to under 380 s at cutoff 2 (Fig. 3A and Table 1). The reason was that at a cutoff value of 1, the whole dataset needed to be calculated for pairwise NW distances with Kmer screening. For the day 3 dataset, a total of 42,257,396 NW distances were calculated at cutoff 1, which took approximately 26,260 s (single CPU thread). With the cutoff value of 2, only the abundant group, which made up less than 30% of the initial read number, was calculated, meaning that the number of pairwise NW alignments was reduced to ∼9% of the initial number. For the day 3 dataset, only 1,612,237 NW distances were calculated at cutoff 2 (3.8% of that at cutoff 1), and only 1,008 seconds (single CPU thread) were used for this step. In comparison, the ESPRIT and SLP-PW-AL methods require more than 200 times as long as TSC. In addition, because the computation time increases geometrically, the TSC pipeline saves even more computing resources than ESPRIT and SLP-PW-AL for larger datasets.

In addition to saving computing time, TSC also significantly reduced the peak memory usage for AL. To perform AL clustering, a distance matrix needs to be loaded into the memory, which is different from the hcluster for CL [11]. However, as the distance matrix file increases geometrically with the sequence number, the peak memory usage becomes a bottleneck for analyzing large datasets. By dividing tags into two groups, we were able to greatly reduce the tag number in the abundant group; therefore, the peak memory usage for TSC was an order of magnitude lower than that of other AL methods (Table 1). Furthermore, the cutoff value could further tune the peak memory usage because the higher the cutoff value, the smaller the intermediate-distance matrix file.

### TSC results for CL/AL/SL merge with increasing cutoff values

**Figure 4:**
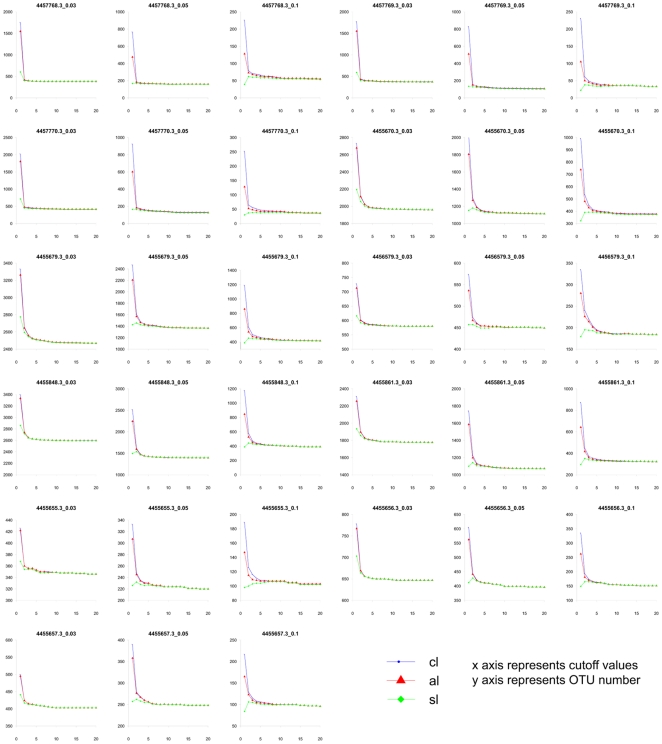
TSC clustering of 11 datasets at distances of 0.03, 0.05, and 0.10. All datasets were downloaded from MG-RAST. 4455655.3, 4455656.3, 4455657.3, 4455670.3, 4455679.3, and 4456579.3 were V2 fragments from soil; 4455848.3 and 4455861.3 were V2 tags from tundra communities; 4457768.3, 4457769.3, and 4457770.3 were V4 tags from human-associated habitats.

One interesting phenomenon we observed was that the clustering results for the three algorithms tended to merge as the cutoff value for defining the two groups increased. At a cutoff value of 1, TSC-CL, AL, and SL showed significantly different OTU estimations (Fig. 2 and 3), whereas these three lines quickly merged from cutoff value 2 to 3. This trend was not unique to the clone43_97up and Costello day 3 datasets. We further analyzed 10 datasets that were determined using either the 454 or the Illumina platform from environmental or human microbiome samples at distances of 0.03, 0.05 and 0.1, and all of them showed the merging of CL, AL, and SL results with increasing TSC cutoff value (Fig. 4). These results indicate that the abundant tags were grouped into similar numbers of OTUs using any of the three algorithms, whereas the significantly different OTU numbers among CL, AL, and SL in previous reports [13] were mainly caused by the rarest tags, mostly singletons and doubletons, similar to what was observed at the cutoff of 1.

Rare sequences receive much attention in both clustering algorithm and microbial diversity studies. With next-generation sequencing techniques, researchers have found an unexpectedly large number of rare species in various environments [4]. However, further studies demonstrated that pyrosequencing errors and PCR mutations contributed substantially to the large number of rare tags [13], [18], [19], [21]. For instance, Quince et al. found that most of the remaining error tags after denoising were singletons [17]. Additionally, in the clone43_97up dataset, all rare sequences were ‘noise’ because there were only 43 templates in the initial PCR cocktail. The best way to mitigate the effect of ‘noise’ sequences while maintaining the true rare biosphere is an intriguing topic [13], [17].

In our TSC pipeline, we presumed that the distance between a ‘noisy’ rare sequence and its parent tag was within a certain distance (such as 0.03). Accordingly, we first clustered the abundant group using highly accurate methods. Subsequently, we merged rare tags into these abundant OTUs if they fit the distance to any tags in the abundant OTUs, thereby minimizing the ‘noise’ effect, as demonstrated in the clone43_97up result. It is possible that diversity could be under-evaluated in the TSC pipeline, e.g., if some rare tags within 0.03 distances to abundant OTUs came from true rare species or if many real rare sequences were linked together using the SL algorithm. According to our analysis, even with this underestimation risk, a large fraction of singleton or doubleton OTUs were retained in the TSC results (Table S1), indicating that a highly diverse rare biosphere could be characterized with the TSC pipeline.

Even though the TSC pipeline can reduce the noise effect from rare tags, the method is not a denoising program. We recommend using Ampliconnoise [17] and Uchime [22] to screen pyrosequencing errors and chimeras before performing the clustering because these types of error sequences with large differences from their parent tags cannot be removed by the pipeline.

Compared with the other methods, TSC (cutoff 3) obtained relatively conservative OTU numbers for the Costello day 3 dataset (Table 1). Although there is concern about the spurious OTU estimation, this does not imply that a method with a lower number of estimated OTUs is better. Our TSC pipeline achieved a lower estimation by minimizing the contribution of rare tags.

### TSC shows comparable or improved performance for beta-diversity comparisons

**Figure 5:**
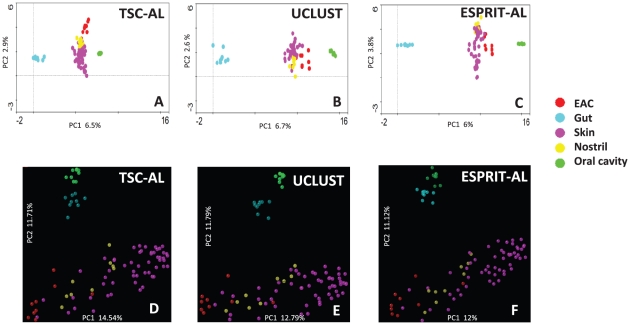
DCA and PCoA analysis of the Costello day 3 dataset using OTU results clustered with different methods. For A–C, we used the abundance data of OTUs clustered using the three methods as input for DCA analysis using Canoco v4.2; For D–F, we picked the most abundant tag from each OTU clustered using various methods as input for calculating unweighted UniFrac distance and used the UniFrac distance for PCoA analysis.

One of the major reasons to group tags into OTUs is to compare the beta diversity of microbial communities to characterize the clustering patterns of communities and to explore their underlying biological explanations. We compared the clustering patterns of Costello day 3 samples using OTUs clustered with various methods (Fig. 5). In general, TSC, UCLUST, and ESPRIT results could separate gut (blue) and oral (green) samples from the others, which was in accordance with the conclusion in the Costello paper [20]. The unweighted UniFrac-based principal coordinates analysis (PCoA) showed similar resolutions for the three approaches and a group of samples from external auditory canal (EAC, red) could also be identified from the skin samples (purple) (Fig. 5 D–F), which was more obvious than the detrended correspond analysis (DCA) results (Fig. 5 A–C). In addition to the Costello dataset, we tested several in-house data (both 454 and Illumina) with TSC and UCLUST, as both methods can analyze datasets with millions of tags. The TSC and UCLUST generally performed similarly for beta-diversity comparison, but sometimes the TSC pipeline worked slightly better than the UCLUST or vis-versa (data not shown). We suggest users to try both methods and select the one showing better results as expectations.

### TSC clusters datasets with over 1 million sequences with high efficiency

**Figure 6:**
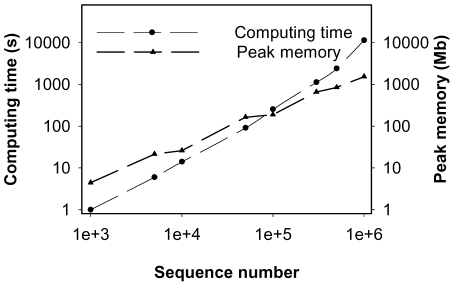
The computing time and peak memory usage of TSC performed on the Costello dataset with a varying number of sequences (1 K–1.07 M).

To evaluate the computational efficiency of TSC, we used the full Costello dataset and its subsamples, varying from 1,000 to 1.07 million sequences. The TSC method showed quasilinear computational complexity, and the peak memory usage of TSC increased linearly with the sequence number (Fig. 6). Four major traits of our TSC pipeline contribute to the improvement of computational efficiency. First, only the abundant tags are clustered using the hierarchical algorithm; therefore, the time required to perform the pairwise NW alignment and the peak memory usage is significantly reduced. Second, the alignment-free preclustering (step 2) treats a large proportion of rare tags, which is more rapid than the alignment methods. Third, a Kmer screening further reduces the number of NW distances that need to be calculated in the second-stage clustering. Finally, TSC enables the use of multiple CPU threads throughout the program, which further reduces the computation time. In brief, the TSC algorithm retains the accuracy of clustering with pairwise NW distances for every tag grouped into OTUs, and it accelerates the speed by minimizing the number of NW distances to be calculated.

The TSC program can be used to analyze both 454 and Illumina data. There is a parameter named −r 454 that uses a penalty value of 0 for continuous extension gaps and end gaps; thus, it calculates these gaps as a single indel, which fits the 454 sequencing error characteristic [18]. In contrast, in the default mode for Illumina (BIPES) reads, each extension and end gap has a penalty value of -1 because the Illumina method tends to have error types of mismatches rather than continuous indels [6]. We used the TSC program to analyze both 454 and Illumina data, and all datasets showed well-clustered OTUs.

In conclusion, the present study demonstrated that rare tags caused both computation and accuracy problems for OTU clustering. Currently, sequencing throughput is increasing rapidly with the advancement of new sequencing techniques, and our study suggests that the characteristics of a sequencing dataset should also be considered for future improvements to bioinformatic analysis pipelines. TSC can analyze large datasets of tags using common personal computers, and we suggest that it will be useful for both large studies and labs without requiring advanced computational resources.

## Supporting Information

Table S1 **Percentage of singleton and doubletons in TSC results of 11 datasets.**
(DOCX)
