# Supplementary material for: Two-Stage Clustering (TSC): A Pipeline for Selecting Operational Taxonomic Units for the High-Throughput Sequencing of PCR Amplicons
Source: PLoS One. 2012 Jan 11;7(1):e30230. doi: 10.1371/journal.pone.0030230 (PMC3256218; doi:10.1371/journal.pone.0030230)
Supplement: Table S1 — Percentage of singleton and doubletons in TSC results of 11 datasets. (DOCX) [file pone.0030230.s001.docx]

Supplementary Table S1.Percentage of singleton and doubletons in TSC results of 11 datasets

| ID | 4455655.3 | 4455656.3 | 4455657.3 | 4455670.3 | 4455679.3 | 4455848.3 | 4455861.3 | 4456579.3 | 4457768.3 | 4457769.3 | 4457770.3 |
| --- | --- | --- | --- | --- | --- | --- | --- | --- | --- | --- | --- |
| S_3_0.03_al | 0.5758 | 0.6997 | 0.6313 | 0.6220 | 0.6452 | 0.6925 | 0.6435 | 0.5008 | 0.6608 | 0.6667 | 0.6608 |
| D_3_0.03_al | 0.1657 | 0.1418 | 0.1373 | 0.1453 | 0.1434 | 0.1368 | 0.1443 | 0.1438 | 0.0528 | 0.0572 | 0.0394 |
| S_3_0.03_cl | 0.5758 | 0.6997 | 0.6313 | 0.6214 | 0.6447 | 0.6925 | 0.6428 | 0.5000 | 0.6608 | 0.6585 | 0.6551 |
| D_3_0.03_cl | 0.1657 | 0.1418 | 0.1373 | 0.1451 | 0.1433 | 0.1368 | 0.1442 | 0.1436 | 0.0528 | 0.0565 | 0.0390 |
| S_3_0.03_sl | 0.5791 | 0.6997 | 0.6344 | 0.6282 | 0.6487 | 0.6935 | 0.6471 | 0.5043 | 0.6675 | 0.6889 | 0.6864 |
| D_3_0.03_sl | 0.1667 | 0.1418 | 0.1380 | 0.1467 | 0.1442 | 0.1370 | 0.1451 | 0.1448 | 0.0533 | 0.0591 | 0.0409 |
| S_3_0.05_al | 0.5342 | 0.6119 | 0.5655 | 0.5295 | 0.5795 | 0.6257 | 0.5801 | 0.4336 | 0.4477 | 0.5075 | 0.4909 |
| D_3_0.05_al | 0.1410 | 0.1619 | 0.1461 | 0.1518 | 0.1427 | 0.1495 | 0.1450 | 0.1460 | 0.0523 | 0.0299 | 0.0364 |
| S_3_0.05_cl | 0.5319 | 0.6105 | 0.5634 | 0.5251 | 0.5736 | 0.6240 | 0.5750 | 0.4317 | 0.4425 | 0.4892 | 0.4709 |
| D_3_0.05_cl | 0.1404 | 0.1615 | 0.1455 | 0.1505 | 0.1412 | 0.1491 | 0.1437 | 0.1453 | 0.0517 | 0.0288 | 0.0349 |
| S_3_0.05_sl | 0.5482 | 0.6148 | 0.5853 | 0.5414 | 0.5954 | 0.6291 | 0.5900 | 0.4393 | 0.4695 | 0.5574 | 0.5294 |
| D_3_0.05_sl | 0.1447 | 0.1627 | 0.1512 | 0.1552 | 0.1466 | 0.1503 | 0.1475 | 0.1479 | 0.0549 | 0.0328 | 0.0392 |
| S_3_0.1_al | 0.4312 | 0.4588 | 0.4000 | 0.3846 | 0.4589 | 0.5000 | 0.4318 | 0.2710 | 0.2206 | 0.3333 | 0.2500 |
| D_3_0.1_al | 0.1651 | 0.1235 | 0.1455 | 0.1725 | 0.1516 | 0.1261 | 0.1337 | 0.1262 | 0.0588 | 0.0222 | 0.0208 |
| S_3_0.1_cl | 0.4052 | 0.4483 | 0.3826 | 0.3675 | 0.4343 | 0.4788 | 0.4167 | 0.2636 | 0.2143 | 0.3061 | 0.2143 |
| D_3_0.1_cl | 0.1552 | 0.1207 | 0.1391 | 0.1648 | 0.1434 | 0.1208 | 0.1290 | 0.1227 | 0.0571 | 0.0204 | 0.0179 |
| S_3_0.1_sl | 0.4563 | 0.4727 | 0.4190 | 0.4209 | 0.4888 | 0.5244 | 0.4572 | 0.2990 | 0.2500 | 0.4054 | 0.3158 |
| D_3_0.1_sl | 0.1748 | 0.1273 | 0.1524 | 0.1888 | 0.1614 | 0.1323 | 0.1416 | 0.1392 | 0.0667 | 0.0270 | 0.0263 |

1. In the first column a_b_c_d, a means singleton (S) or doubleton (D), b means cutoff = 3, c means cluster distance value, d means cluster method.
2. In the first row is the sample ID from MG-RAST.
